# MicroRNA-655 inhibits cell proliferation and invasion in epithelial ovarian cancer by directly targeting vascular endothelial growth factor

**DOI:** 10.3892/mmr.2022.12860

**Published:** 2022-09-26

**Authors:** Ziling Zhao, Shuna Yang, Yan Cheng, Xiuhua Zhao

Mol Med Rep 18: 1878–1884, 2018; DOI: 10.3892/mmr.2018.9090

Following the publication of this paper, it was drawn to the Editors’ attention by a concerned reader that certain of the cell invasion assay data shown in Figs. 2C and 4C were strikingly similar to data appearing in different form in other articles by different authors. Owing to the fact that the contentious data in the above article had already been published elsewhere, or were already under consideration for publication, prior to its submission to *Molecular Medicine Reports*, the Editor has decided that this paper should be retracted from the Journal. The authors were asked for an explanation to account for these concerns, but the Editorial Office did not receive a reply. The Editor apologizes to the readership for any inconvenience caused.

